# Local and free flaps in cutaneous nasal oncologic reconstruction

**DOI:** 10.3389/fonc.2026.1806517

**Published:** 2026-08-18

**Authors:** Rahaf AlThomali, S. Mark Taylor

**Affiliations:** Division of Otolaryngology – Head and Neck Surgery, Department of Surgery, Dalhousie University, Halifax, NS, Canada

**Keywords:** nasal aesthetic subunit, nasal reconstruction, nasolabial flap, oncologic resection, paramedian forehead flap, radial forearm free flap, sinonasal malignancies, timing of reconstruction

## Abstract

Reconstruction of cutaneous nasal defects following resection of sinonasal malignancies remains a complex surgical challenge because of the nose’s intricate anatomy and its critical aesthetic and functional roles. Multiple reconstructive options are available to restore nasal integrity; however, no universally accepted optimal approach exists. This review summarizes contemporary approaches to oncologic nasal reconstruction using local, regional, and free flaps, with emphasis on the aesthetic subunit principle as the foundation of surgical planning. Reconstructive decision-making is guided by defect size, depth, and subunit involvement, as well as patient-specific factors such as comorbidities, prior irradiation, and the anticipated need for adjuvant therapy. Local and regional flaps, including the nasolabial flap and the paramedian forehead flap, are preferred for small to moderate cutaneous nasal defects, whereas free flaps, particularly the radial forearm flap, provide reliable solutions for extensive composite defects. Immediate reconstruction is generally associated with superior aesthetic, functional, and psychosocial outcomes; however, delayed reconstruction may be appropriate in selected cases requiring margin surveillance or additional oncologic assessment. This review aims to provide practical insights into reconstructive decision-making based on the available literature and clinical experience, with the goal of optimizing aesthetic, functional, and oncologic outcomes in patients undergoing oncologic nasal reconstruction.

## Introduction

Sinonasal malignancies represent a rare and diverse group of head and neck cancers with generally poor prognoses, for which surgical resection remains the mainstay of treatment when feasible. These tumors account for 5% of all head and neck cancers, with squamous cell carcinoma representing the most common histologic subtype (1). Sinonasal malignancies most frequently arise in the nasal cavity, maxillary sinus, and ethmoid sinuses (1). The principal goal of treatment is complete tumor excision with histologically clear margins. However, the proximity of the nasal cavity and paranasal sinuses to critical neurovascular structures makes surgical resection particularly challenging (1). Achieving oncologic resection often results in significant tissue loss, requiring meticulous reconstructive planning to restore both nasal form and function while preserving aesthetic and functional integrity (2).

The nose occupies a central position on the face, serving as both a vital aesthetic landmark and a functional structure essential for breathing and olfaction. Its intricate anatomy and dual aesthetic–functional importance make nasal reconstruction technically demanding and socially significant. Even minimal tissue loss can produce significant deformities that lead to psychological and social distress (3). Deformities involving the nasal valve may also impair airflow, further reducing quality of life. Moreover, following cancer treatment, patients often experience heightened expectations for aesthetic outcomes as part of their overall recovery (4).

Nasal reconstruction has a long historical tradition, dating back to ancient India, where Sushruta first described the use of a cheek flap around 600 BC (5). Over centuries, these principles evolved into more sophisticated techniques, culminating in the aesthetic subunit concept introduced by Burget and Menick, which remains a cornerstone of modern reconstructive planning. Currently, local and regional flaps are the mainstay for cutaneous nasal defects, whereas microsurgical free tissue transfer provides additional options for extensive or subtotal nasal reconstructions.

This review aims to summarize contemporary approaches to oncologic nasal reconstruction for cutaneous nasal defects following resection of sinonasal malignancies. It focuses on defect assessment, the role of the aesthetic subunit principle, appropriate flap selection based on defect size and complexity, and the optimal timing of reconstruction. Our objective is to provide a concise, clinically oriented overview reflecting commonly utilized and versatile reconstructive options in our practice, rather than an exhaustive review of all described flap techniques.

## Materials and methods

This review was conducted using a targeted literature search in PubMed to identify relevant studies on local and free flap techniques in oncologic nasal reconstruction of cutaneous nasal defects.

The literature search was performed between October 2025 and January 2026 and used combinations of the following keywords: *sinonasal malignancies*, oncologic *nasal reconstruction*, *cutaneous nasal defects*, *nasal aesthetic subunit*, *local flaps*, *nasolabial flap*, *paramedian forehead flap*, *radial forearm free flap*, *free tissue transfer*, and *timing of reconstruction*.

Given the narrative nature of this review, no predefined inclusion or exclusion criteria were applied. Instead, articles were selected based on their clinical relevance, methodological quality, historical significance, and contribution to reconstructive principles and outcomes. Priority was given to peer-reviewed studies and widely cited publications that have shaped current reconstructive practice. Additional references were identified through manual review of reference lists.

Formal systematic review protocols were not employed. Rather, the selected literature was synthesized with the authors’ clinical experience to provide a concise, practice-oriented overview of reconstructive strategies in oncologic nasal reconstruction.

Written informed consent was obtained from all patients for publication of the clinical images included in this manuscript.

### Defect evaluation

Nasal anatomy can be conceptualized as three distinct structural layers: the cover, comprising the skin, subcutaneous tissue, and muscle; the framework, which includes cartilage and bone; and the internal lining, formed by the vestibular skin and nasal mucosa (6). Comprehensive evaluation of nasal defects requires careful assessment of these layers, as well as consideration of the surrounding tissue quality, residual or recurrent tumor presence, and prior scars or radiation changes (6).

The most important parameters guiding reconstructive planning are the size, depth, and location of the defect. Small defects measuring less than 1.5 cm in diameter can often be managed with primary closure, local flaps, or full-thickness skin grafts (FTSG). Medium-sized defects between 1.5 and 2.5 cm may require local or regional flaps, and in select cases, FTSGs may suffice. Large defects greater than 2.5 cm generally necessitate regional or free flap reconstruction. Although no universal consensus in the literature exists regarding these thresholds, such classification assists in systematic preoperative planning and selection of appropriate techniques (2, 7).

### Nasal subunits and reconstructive principles

The aesthetic subunit principle remains central to nasal reconstruction. Gonzalez-Ulloa et al. first described the concept of dividing the face into distinct aesthetic units, which Burget later refined by subdividing the nose into nine subunits: the dorsum, sidewalls, tip, columella, alae, and soft triangles (8). Reconstruction of entire subunits rather than partial replacement yields more harmonious and less conspicuous results, particularly when defects involve more than 50% of a subunit (8). Quantitative studies supported this approach (9), demonstrating that complete subunit replacement of the nasal tip or dorsum provides superior aesthetic outcomes compared with partial repair.

When defects cross multiple aesthetic units, such as those extending from the alar sulcus onto the cheek, each region should be reconstructed separately to preserve natural contours and avoid distortion (10). Reconstructive decision-making is guided by an integrated assessment of defect size, depth, nasal subunit involvement, and patient-specific factors, which together inform the choice of technique and timing of intervention. Successful reconstruction requires restoring the nasal lining, re-establishing structural support with cartilage or bone grafts, and providing external skin coverage that matches surrounding tissue in color, thickness, and texture (11). Secondary technical refinements, including contour correction, flap thinning, precise edge approximation, and strategic placement of cartilage grafts, particularly at the alar margin, are essential for achieving optimal results (12, 13). Although the subunit principle is widely accepted, it can be selectively modified. Small dorsal defects, for example, may be better managed with local flaps or skin grafts rather than a full-thickness forehead flap to avoid contour and thickness mismatch (12).

Oncologic considerations such as margin status, anticipated radiotherapy, and the quality of the recipient bed play a decisive role in flap selection (6). In this context, local flaps are generally favored for smaller, well-vascularized defects, whereas microsurgical free flaps are reserved for extensive, composite, or compromised defects requiring multilayer reconstruction. The timing of reconstruction; immediate versus delayed, must also be tailored according to oncologic safety and patient-specific factors. Postoperative evaluation should include assessment of airway patency, donor-site morbidity, and the aesthetic quality of the reconstruction to ensure comprehensive recovery.

### Common locoregional flaps for nasal reconstruction

#### Nasolabial flap

The nasolabial flap is one of the most versatile options for small to moderate nasal defects. First described by Dieffenbach, it is a random-pattern flap that relies on the rich vascular network of the nasolabial region, primarily supplied by branches of the facial, angular, and infraorbital arteries (14). The medial cheek skin provides excellent color and texture match, making the nasolabial flap particularly suitable for reconstructing defects of the nasal ala and lower sidewall (15). Its robust vascularity allows it to support free cartilage grafts in complex reconstructions (16), and donor-site morbidity is minimal because the scar is easily concealed within the natural nasolabial crease.

This flap can be designed as either a transpositional or interpolated flap. The transpositional variant provides single-stage reconstruction, with the flap base positioned adjacent to the defect, typically in a superiorly based configuration to optimize pedicle mobility or inferiorly based to minimize the risk of pincushioning. This approach offers simplicity but may occasionally cause distortion or partial effacement of the alar–facial sulcus (15).

The interpolated nasolabial flap, typically performed in two stages, preserves the natural contour of the alar–facial groove and may utilize either a cutaneous or subcutaneous pedicle. The second stage pedicle division is usually completed about three weeks after initial flap elevation. When a subcutaneous pedicle is chosen, the required wider dissection increases the risk of facial nerve branch injury (14), but this risk can be minimized by maintaining planes above the facial musculature.

Moreover, this flap can be adapted for columellar reconstruction by folding the tissue and incorporating a cartilage strut graft for structural support. In full-thickness nasal defects, it can also serve as a reliable option for internal lining when other options are unavailable (16). Limitations include its restricted tissue reservoir for large defects and reduced reliability in previously irradiated or scarred fields.

#### Paramedian forehead flap

The paramedian forehead flap (PFF) remains the cornerstone of nasal reconstruction for large and complex defects involving one or more aesthetic subunits (17). This axial-pattern interpolated flap is based on the supratrochlear artery and is typically performed in two stages. Historically described in ancient Sanskrit texts and later refined by Millard, the paramedian variant allows greater mobility and a narrower pedicle, establishing it as the preferred method for total or subtotal nasal reconstruction (18).

The pedicle, typically about 10 mm wide, is centered on the supratrochlear artery, located approximately 1.8–2.2 cm lateral to the midline. The supratrochlear artery emerges from the orbit, travels superficial to the corrugator muscle and deep to the orbicularis oculi, pierces the frontalis muscle roughly 2–3 cm above the orbital rim, and then proceeds in a subcutaneous plane (19).

Flap elevation proceeds in the subgaleal plane with some surgeons transitioning to a subperiosteal plane near the pedicle to protect the vascular supply. Our preferred depth of harvest remains in the subgaleal plane throughout the dissection, with medial and lateral division of the corrugator muscle. Donor sites can usually be closed primarily, although secondary healing may be required for larger defects (18). Distal thinning should be performed judiciously, particularly in smokers to avoid compromising vascularity (19).

The standard reconstruction is carried out in two stages: the flap is first elevated and inset into the nasal defect, followed approximately three weeks later by pedicle division. Both single-stage and three-stage modifications have been described to accommodate specific clinical scenarios.

Despite requiring multistage procedures, resulting in a forehead donor-site scar, potential flap thickness necessitating secondary thinning, and a longer operative time, it remains a highly reliable reconstructive option (20). It offers predictable vascularity, versatility, excellent color match, and can be safely performed even in elderly or comorbid patients. Immediate PFF reconstruction enables early restoration of nasal form and function and facilitates timely initiation of adjuvant therapy; however, close coordination with oncologic margin assessment is essential to ensure oncologic safety.

### Free flaps in nasal reconstruction

For extensive or composite nasal defects, the radial forearm free flap (RFFF) provides a versatile and reliable reconstructive option. Its thin, pliable, and well-vascularized tissue allows precise three-dimensional restoration of both the internal lining and external cover (21, 22). It is particularly valuable in oncologic reconstruction when local tissues have been depleted by tumor excision, prior radiation, or previous failed reconstruction (23). The RFFF’s long pedicle and robust perfusion make it suitable even in compromised recipient beds, and structural components such as tendon or bone can be harvested to restore framework support.

Clinical outcomes consistently demonstrate the reliability of the RFFF in complex nasal reconstruction. Walton et al. (24) and Ettl et al. (25) reported successful use of this flap for intranasal lining reconstruction after prior flap failures, emphasizing its vascular robustness, while Burget described excellent outcomes using multi-paddle configurations to reconstruct individual subunits and improve airway patency. Winslow et al. (26) further expanded its application to total nasal reconstruction using de-epithelialized flaps, highlighting their long-term stability and resistance to contracture.

In the setting of total nasal reconstruction, the principles described by Menick emphasize a multistage approach based on restoration of three essential components: internal lining, structural support, and external skin cover (27). This method typically involves staged reconstruction, beginning with establishment of a well-vascularized lining, followed by placement of cartilage or bony framework for durable support, and culminating in soft tissue coverage, most commonly using a PFF. Subsequent stages focus on flap division, contour refinement, and optimization of both aesthetic and functional outcomes. These principles underscore the importance of sequencing and tissue specificity in achieving durable nasal reconstruction and are applicable regardless of whether local or free tissue transfer is utilized.

Although the technique requires microsurgical expertise and may result in visible donor-site morbidity, the RFFF remains one of the most effective solutions for restoring nasal form and function when regional options are unavailable.

### Timing of reconstruction

The timing of nasal reconstruction remains a key consideration in oncologic surgery. Immediate reconstruction offers the advantages of a single-stage procedure, early restoration of appearance and airway function, and improved psychological recovery. However, this approach can pose challenges, including uncertain margin status, prolonged operative time, and the need for close coordination with radiotherapy.

Delayed reconstruction, in contrast, allows for histologic confirmation of clear margins, optimization of patient health, and completion of adjuvant therapy before definitive reconstruction in selected cases. These benefits must be balanced against prolonged disfigurement, increased risk of wound contraction, and potential psychosocial distress.

The optimal timing of reconstruction depends largely on tumor biology and clinical context. Basal cell carcinoma, which is typically managed with Mohs micrographic surgery, often allows safe immediate reconstruction. Squamous cell carcinoma, being more aggressive and prone to deeper invasion, may warrant delayed reconstruction, sometimes for up to one year, to allow adequate surveillance for local recurrence. Performing immediate reconstruction with large flaps in SCC cases may obscure residual disease and delay detection of recurrence (1). This decision should be balanced against patient-specific factors and the aggressive biological behavior of the sinonasal malignancy.

Current evidence from several comparative retrospective studies has evaluated the perioperative and long-term outcomes of immediate versus delayed reconstruction following oncologic resection in the head and neck region. Overall, the findings have been heterogeneous, and differences in study design, sample size, tumor site, and reconstructive technique limit direct comparison and preclude a definitive consensus. Consequently, the timing of reconstruction should be individualized, taking into account oncologic factors such as margin status and the anticipated need for adjuvant therapy, as well as patient-specific variables including comorbidities, recipient bed quality, defect complexity, and institutional expertise. Therefore, when oncologic safety is clear and patient factors permit, immediate vascularized reconstruction is generally preferred, as it facilitates efficient restoration of nasal form and function while maintaining oncologic integrity.

### Evidence summary and future directions

Existing evidence demonstrates that the PFF provides highly reliable outcomes for extensive external nasal defects with minimal failure rates, while the nasolabial flap remains an effective option for smaller defects with limited morbidity. In our practice, the nasolabial flap is preferred for isolated alar defects, whereas the PFF is generally used for defects involving two or more nasal subunits. Free flap reconstruction, particularly using the RFFF, is advantageous for large composite defects or cases involving compromised local tissues. Vascularized flaps also demonstrate superior tolerance to radiotherapy. The timing of reconstruction significantly influences outcomes: immediate vascularized reconstruction promotes early functional recovery and integration with adjuvant therapy, whereas delayed reconstruction may be more appropriate when oncologic uncertainty persists. Donor-site morbidity, patient counseling regarding multi-stage procedures, and clear communication of expected functional and aesthetic outcomes are essential components of comprehensive care.

Most published data are retrospective, and standardized patient-reported outcome measures (PROMs) are lacking. Future studies should focus on prospective comparisons between flap types and reconstruction timing, incorporation of validated PROMs, cost-effectiveness analyses, and refinement of flap techniques tailored to nasal subunits.

## Conclusion

Oncologic nasal reconstruction is grounded in the aesthetic subunit principle and requires a detailed understanding of the complex nasal anatomy, differing tissue characteristics among nasal subunits, and individualized surgical planning. The central objectives are complete tumor resection with clear margins and the restoration of optimal aesthetic and functional outcomes. Each defect presents unique challenges, and reconstructive strategies must be customized according to oncologic safety, patient factors, and aesthetic demands. Immediate reconstruction is preferred whenever feasible, while delayed reconstruction remains appropriate in select cases. Optimizing functional, aesthetic, and oncologic outcomes depends on the interplay between timing, flap selection, and patient-specific factors. Continued research is needed to refine evidence-based reconstructive algorithms and better integrate patient-centered outcome measures into oncologic nasal reconstruction practice.
